# Immediate Effects and 1‐Year Maintenance of a Voice Education Program for Older Adults

**DOI:** 10.1111/1460-6984.70179

**Published:** 2026-01-07

**Authors:** Estella P.‐M. Ma, Ally O.‐M. Ng, Crystal W.‐N. Yuen

**Affiliations:** ^1^ Voice Research Laboratory, Unit of Human Communication, Learning, and Development, Faculty of Education University of Hong Kong Hong Kong Hong Kong

**Keywords:** aging voice, presbyphonia, prevention, vocal hygiene, voice care knowledge

## Abstract

**Background:**

Our voice can deteriorate with ageing. Vocal hygiene is useful and effective in maintaining a healthy voice regardless of age.

**Aim:**

This study investigated the immediate effects and 1‐year maintenance of a voice education program on promoting voice care for older adults.

**Methods and Procedures:**

Thirty‐five participants aged over 55 were recruited. They participated in a weekly 1‐h voice education workshop for four consecutive weeks. Their responses on the 17‐statement questionnaire were collected before the program began, immediately after, and 1 year after the program was completed to assess change in their voice care knowledge. Upon completion of the program, their satisfaction with the program was reflected by an 8‐item satisfaction survey. A semi‐structured group interview was conducted to investigate their attitudes towards implementing voice care practice.

**Outcomes and Results:**

Immediate significant improvements in voice care knowledge were shown, with fair maintenance 1 year after the program had completed. Most participants showed satisfaction towards the program. The analysis of verbatim transcripts revealed participants’ positive attitudes towards the implementation of voice care practice in their daily lives. Facilitators in the program that contributed to their improvements in voice care knowledge were identified. Barriers that hindered their learning were also identified, and the relevant solutions were proposed to address the barriers.

**Conclusion and Implications:**

The results provide empirical data to support the use of a voice education program to enhance older adults’ voice care knowledge.

**WHAT THIS PAPER ADDS:**

*What is already known on this subject*

- Our voice can deteriorate with ageing. Vocal hygiene is useful and effective in maintaining a healthy voice regardless of age.

*What this paper adds to existing knowledge*

- We found that participating older adults were not well‐equipped with voice care knowledge. Before training, less than half of the participants could correctly identify the negative factors such as throat clearing, whispering and breathing through mouth. The study offers empirical data on the immediate effects and 1‐year maintenance of a voice education program on older adults’ voice care knowledge.

*What are the potential or actual clinical implications of this work?*

- This paper suggests that a voice education program is useful to improve voice care knowledge in older adults.

## 1 Introduction

Population ageing has become a prominent concern worldwide. With reference to the World Health Organization (WHO) (2023), the population of older adults aged 65 years or above is estimated to surpass the youth group under the age of 15 by 2024 in developed countries. Health professionals have increasingly placed much focus on common health conditions associated with ageing in recent years. In the older population, the conditions of laryngeal structures and phonatory physiology decline with advancing ageing. The vocal mechanism is influenced by age‐related changes in the larynx including the ossification and calcification of laryngeal cartilages (de la Grandmaison et al. 2003; Turk and Hogg 1993), laryngeal muscle atrophy (Martins et al. 2015) and degeneration of mucosal glands (Gracco and Kahane 1989). These age‐related changes can lead to vocal fold stiffness and the inadequate alignment of vocal folds can cause irregular vibratory behaviours and glottic incompetence (Ramig et al. 2001). The term presbylarynx has been used to describe the structural and physiological changes in the larynx due to ageing. The deterioration in vocal qualities and voice‐related quality of life associated with presbylarynx is called presbyphonia.

The prevalence of voice disorders in the older population is reported across different countries. A recently published systematic review by Wang and colleagues (Wang et al. 2023) examined 13 published studies on the prevalence of voice disorders in individuals aged 60 or above. The pooled prevalence rate was reported to be 18.8% in this age cohort. Participants in these 13 studies were recruited from the community (nine studies) or institutional settings such as hospitalised wards and nursing homes (four studies). Voice disorders were defined using different criteria, including the use of general questions about one's subjective self‐perception of voice problems, and questionnaires such as Voice Handicap Index (VHI) (Jacobson et al. 1997). Regarding the prevalence rate of presbyphonia among older adults with voice disorders, another systematic review by Chang and colleagues (Chang et al. 2023) reported a pooled prevalence rate of 17.8%. Ten out of 11 studies reported the use of laryngoscopy or stroboscopy to identify the presence of presbyphonia in participants.

Older adults with presbyphonia demonstrate poorer voice quality than those with healthy voices (Crawley et al. 2018). They self‐report experiencing breath shortage with speaking, difficulties speaking loudly in noise and an occasional need to repeat themselves during conversation. These difficulties have significantly diminished their voice‐related quality of life by reducing communication effectiveness, limiting their social participation and causing emotional problems such as frustration (Golub et al. 2006; Roy et al. 2007). To address these substantial effects caused by irreversible ageing, voice education and training is recommended as a preventive measure (Mezzedimi et al. 2017). Voice education enhances older adults’ understanding on laryngeal mechanism, vocal hygiene concepts and use of facilitative techniques such as relaxation exercises aiming at improving one's voice quality (Galluzzi and Garavello 2018). By acquiring the skills and knowledge, older adults with presbyphonia may know how to better manage age‐related vocal changes and to maintain vocal health.

In recent years, numerous studies have reported supportive evidence of voice education program to promote voice care knowledge in professional voice users such as teachers (Bolbol et al. 2017; Faham et al. 2017; Porcaro et al. 2021; Richter et al. 2016), professional singers (Pomaville et al. 2020; Zuim et al. 2021) and journalists (Rodero et al. 2018). However, limited literature is present to demonstrate the effectiveness of voice education programs targeting the ageing population. A case control study (Berg et al. 2008) involved 25 participants aged 60 or above who were diagnosed with presbyphonia. The treatment group (*n* = 19) received vocal education sessions about learning laryngeal physiology and vocal exercises. The control group (*n* = 6) did not receive any treatment. The efficacy of the treatment was evaluated using the voice‐related quality of life (VRQOL) questionnaires taken before, immediately after and at least 2 months after the treatment completed. Their results revealed immediate significant improvement in voice‐related quality of life, which was maintained after an average of 5.1 months. However, the study's validity was limited due to the lack of clearly specified treatment details. In another study (Çiyiltepe and Şenkal 2017), 91 participants aged from 50 to 91 years were recruited. All the participants reported presence of voice problems either associated with ageing, chronic or neurological diseases. Participants were assigned to either receive vocal hygiene therapy (*n* = 75) or symptomatic therapy (*n* = 15). Participants assigned to the vocal hygiene therapy learned about vocal hygiene concept, postural modification, breathing supporting exercises, modified resistance exercises and lingual strengthening exercises. The symptomatic therapy involved a combination of voice programs such as Lee Silverman Voice Therapy. The effectiveness of voice therapy across different age groups was assessed through perceptual and acoustic measurement as well as the questionnaire of Voice Handicap Index (VHI) administered before and after the therapy was completed. Significant improvements in voice quality and reduction in the VHI scores were reported in 50–59‐ and 60–69‐year groups only, except for 70–91‐year group. However, this study was limited by the absence of long‐term follow‐up assessment.

To fill the research gap, the objective of this study was to examine the immediate training effects and 1‐year maintenance of a voice education program on enhancing older adults’ voice care knowledge. The change in voice care knowledge pre‐ and post‐training would be compared to assess the immediate treatment effect, while the follow‐up data obtained 1 year after the program was completed would be used to examine the maintenance effect. Participants’ satisfaction towards the program and attitudes towards implementation of voice care practice would be documented by a satisfaction survey and a semi‐structured group interview, respectively. It was hypothesised that the voice education program would be effective in improving older adults’ voice care knowledge.

## 2 Methods

This study had attained ethics approval from the Human Research Ethics Committee of the University of Hong Kong (Approval number: EA210375). Informed consent was collected from all participants before they joined the program.

### 2.1 Participants

Thirty‐five participants (13 males and 22 females) with mean age of 71.9 years (standard deviation = 6.78; range = 55 to 85 years) were recruited from two community centres in Hong Kong. An information flyer about the project was sent to all registered members of the community centres. Members who were interested in the program then signed up on a voluntary basis. The inclusive criteria included: (1) age 55 or above, (2) Cantonese language proficiency and (3) full attendance of all sessions in the program. No screening was conducted for the presence of voice disorders. Nevertheless, participants would be excluded from the program if they had attended any training or workshop related to vocal hygiene before participating in this study.

### 2.2 Procedures

#### 2.2.1 Pre‐Training Testing

Before the program started, a 17‐item questionnaire assessing voice care knowledge was distributed to all participants. Each participant had to determine if the items representing voice use behaviours were either ‘beneficial’ or ‘harmful’ to one's voice. To avoid random guessing, an option of ‘uncertain’ was provided for each item.

#### 2.2.2 Voice Education Program

An educational voice program called ‘ Intergeneration Voice Buddy Program’, consisting of four consecutive weekly group training sessions, was conducted. Table 1 lists the details of the program. Each session lasted for one hour. All sessions were facilitated by a certified speech and language therapist with experience in geriatric voice care. Various topics, including age‐related changes in laryngeal anatomy and phonation physiology, common vocal pathologies associated with ageing, vocal hygiene concepts, vocal facilitative techniques of humming and sighing, and their relevance to voice production, were addressed across different sessions. At the end of each session, participants were given handouts and daily practice materials to recap and revisit the materials covered in the session. In addition, a laboratory visit to the Voice Research Lab at the University of Hong Kong was arranged at the end of the program. The laboratory visit aimed to enhance participants’ interest towards voice protection and to make the voice education program more memorable to them. During the visit, participants gained hands‐on experience with aerodynamic and acoustic instrumentation to get a better understanding of their own voice.

**TABLE 1:** Details of the voice education program.

Session	Content
1	Age‐related laryngeal anatomy and phonation physiology; common vocal pathologies associated with ageing.
2	Revision and elaboration of materials presented in the previous session; introduction and practice of diaphragmatic breathing; introduction and practice of humming exercise (up to disyllabic level).
3	Revision and elaboration of materials presented in the previous session; introduction of vocal hygiene concepts; introduction and practice of relaxation exercises.
4	Revision and elaboration of materials presented in the previous session; introduction and practice of maximum phonation exercises; introduction and practice of the vocal facilitative technique of sighing (up to disyllabic level).
5	Visit the Voice Research Laboratory at the University of Hong Kong.

#### 2.2.3 Post‐Training Testing

Each participant was asked to complete two sets of questionnaires for assessing voice care knowledge and their satisfaction towards the program immediately after the program was completed. In the satisfaction survey, each participant was asked to rate their degree of satisfaction towards the program in terms of its content and activity planning in seven statements using a 5‐point scale. Each participant was asked to complete the questionnaire assessing voice care knowledge, as well as the maintenance data, 1 year after joining the program.

#### 2.2.4 Semi‐Structured Group Interview

Focus group interviews were conducted 1 year after the completion of the program to evaluate whether and how participants’ attitudes towards implementing voice care practice changed after joining the program. All participants who attended the voice education program were invited to participate in the interview. Consent was then obtained from those who replied ‘ yes ’ to participate. Participants who consented to participate were included in the focus group interview. Three focus groups were conducted, with two to three participants in each group. The principal researcher conducted the interview using the same topic guide, focusing on four areas, namely (1) the implementation of voice care knowledge, (2) learning facilitators, and (3) learning barriers throughout the program, as well as (4) suggestions to improve the program. Table 2 lists the example questions in each topic area.

**TABLE 2:** Interview questions and relevant topic areas.

Topic area	Example questions
Implementation of voice care knowledge	What did you learn from our program? How did you apply the voice care knowledge in daily life?
Learning facilitators	How did the program facilitate your learning on voice care knowledge? How did the program facilitate your application of vocal facilitative techniques or exercises?
Learning barriers	Have you experienced any difficulties or challenges in learning the voice care knowledge in the program? Have you experienced any difficulties or challenges in practising vocal facilitative techniques or exercises in the program?
Suggestions	Do you have any suggestions to improve our program?

The average duration of interviews was 36 min (SD = 7.55; range = 28 to 43 min). All the interviews were audio‐recorded using a smartphone (iPhone 15; Apple Inc., One Apple Park Way, Cupertino, CA, USA) and transcribed verbatim in Chinese.

### 2.3 Data Analysis and Statistical Analysis

The IBM SPSS statistical software (version IBM Corp., Armonk, NY, USA) was used to conduct the statistical tests. The repeated measures analysis of variance (ANOVA) was used to evaluate the effectiveness of the program on improving voice care knowledge for participants. Participants’ voice care knowledge scores (dependent variable) collected at the three time points, namely before, immediately after and 1 year after the program was completed (independent repeated variable), were analysed and compared. With the voice care knowledge score being the primary outcome variable, the score was calculated by adding results of 17 statements in the questionnaire. In each statement, 1 point was given for a correct answer while 0 point was given for an incorrect response. The score was then standardised to 100 for conducting statistical analysis. In addition, to ensure accurate results interpretation of repeated measures ANOVA, Mauchly's test of sphericity was employed to determine if the assumption of sphericity held, indicating that variances of differences between all the time points are equal. If Mauchly's test revealed a violation of compound symmetry assumption indicated by *p* < 0.05, adjustment of degrees of freedom using Huynh‐Feldt epsilon correction would be used (Blanca et al. 2023).

To obtain a more holistic view of the program's effectiveness, participants’ voice care knowledge score across three time points was compared. The percentage changes in (1) participants’ pre‐ and post‐ scores as well as (2) participants’ post‐ and maintenance scores would be reported, respectively. Additionally, the percentage of participants who showed satisfaction with the program after joining would be presented.

In addition, a qualitative content analysis was conducted on the interviews. The qualitative data analysis software of NVivo (version 14; QSR International, Burlington, MA, USA) and Microsoft Word software program were adopted to manage the data. Following the guideline proposed by Graneheim and Lundman (2004), the principal researcher and partner researcher first read through the transcripts to familiarise themselves with the content, and then they identified meaningful units within the phrases. These meaningful units were shortened, condensed and labelled with codes, which were later sorted into semantically related sub‐themes and themes. The trustworthiness of the findings could be ensured by achieving three evaluative measures of credibility, dependability and transferability (Graneheim and Lundman 2004). Credibility refers to the reasonableness and consistency of data interpretation to reflect participants’ views (Polit and Hungler 1999). Throughout the interview, member checking was conducted by the principal researcher to provide an oral summary of key findings to corresponding participants for additional clarification. In the data analysis process, peer checking was conducted. The researchers held regular team meetings to constantly examine and modify the existing codes and emergent themes to accommodate new insights. The process of seeking agreement among the researchers facilitated identification of the most representative and relevant segments from the transcripts to generate codes and themes. Moreover, dependability refers to the degree of data consistency over time (Lincoln and Guba 1985). During the interview, the same set of questions was asked for all participants to avoid inconsistency in data collection. While the rationales behind researchers’ analytic decisions in code selection and theme generation were fully documented by an ‘audit trail’ (Koch 1994), the regular team meetings involving two researchers were recorded to ensure transparency of decision‐making process. Lastly, the transferability refers to the extent to which the findings were applicable to a greater sample (Polit and Hungler 1999). It was facilitated by providing a clear description of data collection and analysis, and was supported by quotation of representative texts from the transcripts. Table 3 shows a summary of strategies adopted to fulfil each criterion in the study.

**TABLE 3:** Criteria of evaluating trustworthiness (based on Graneheim and Lundman 2004).

Criteria	Explanation of the criteria	Strategies adopted
Credibility	Making reasonable and consistent interpretation of the data	Conducting peer checking and member checking Quoting representative texts from the transcripts
Dependability	Maintaining consistency in data collection over time and ensure scrutinization of final research outcomes	Asking all participants the same set of questions Documenting the research process through ‘audit trail’ and recording
Transferability	Transferring the findings to wider sample	Giving clear and distinct description of data collection and analysis Quoting representative texts from the transcripts

## 3 Results

### 3.1 Quantitative Results

Tables 4 and 5 describe the percentage of responses for voice care knowledge questionnaire and satisfaction survey, respectively. In total, 34 out of 35 participants (13 males and 21 females) with mean age of 71.8 years (SD = 6.84; range = 55 to 85 years) completed the voice care knowledge questionnaires before, immediately after and 1 year after the training. The mean pre‐training accuracy score was 71.3% (SD = 26.40; range = 20.6 to 100.0%). The mean post‐training accuracy score was 81.3% (SD = 20.66; range = 41.2 to 100.0%). The mean maintenance training accuracy score obtained 1 year after the training was 80.3% (SD = 20.13; range = 47.1 to 100.0%). The result of Mauchly's Test of Sphericity was not significant (Mauchly's *W* = 0.930, df = 2, *p* = 0.313), indicating the variances in the difference of voice care scores between all the time points were equal. The data did not violate the assumption of sphericity. The results of the ANOVA found significant difference in voice care knowledge accuracy score before, immediately after and 1 year after training (*F*[2, 66] = 9.98, *p* < 0.001, partial *η^2^* = 0.23). Post‐hoc tests with Bonferroni corrections revealed a significant increase in the post‐training accuracy scores collected immediately after training (*p* < 0.001). Given that the maintenance accuracy scores collected 1 year after training were significantly higher from that obtained at pre‐training (*p* = 0.006), the maintenance scores remained similar and did not significantly differ from the post‐training scores collected immediately after training (*p* = 1.00).

**TABLE 4:** Distribution of responses (in percentage) for voice care knowledge questionnaire taken before, after and 1 year after training (*N* = 34).

Questionnaire item	Pre‐training			Post‐training			Maintenance		
	Positive factor	Negative factor	Not certain	Positive factor	Negative factor	Not certain	Positive factor	Negative factor	Not certain
Positive factors									
1. Sufficient sleep	**88.2**	2.9	8.8	**100.0** ^a^	0.0	0.0	**100.0** ^b^	0.0	0.0
2. Drink more water	**94.1**	0.0	5.9	**97.1** ^a^	0.0	2.9	**100.0** ^b^	0.0	0.0
3. Maintain good posture	**70.6**	0.0	29.4	**100.0** ^a^	0.0	0.0	**91.2**	2.9	5.9
4. Do more stretching exercises	**85.3**	0.0	14.7	**100.0** ^a^	0.0	0.0	**91.2**	0.0	8.8
5. Maintain in a good mood	**100.0**	0.0	0.0	**100.0** ^a^	0.0	0.0	**100.0** ^b^	0.0	0.0
6. Avoid chatting in noisy environments	**38.2**	47.1	14.7	**41.2** ^a^	52.9	5.9	**58.8** ^b^	41.2	0.0
*Overall average (positive factors)*	***79.4***	*8.3*	*12.3*	***89.7***	*8.8*	*1.5*	***90.2***	*7.4*	*2.5*
Negative factors									
7. Chronic coughing	0.0	**100.0**	0.0	0.0	**100.0**	0.0	5.9	**91.2**	2.9
8. Laugh/Cry loudly	11.8	**82.4**	5.9	20.6	**73.5**	5.9	17.6	**67.6**	14.7
9. Drink coca cola, coffee and tea	2.9	**58.8**	38.2	2.9	**85.3** ^a^	11.8	2.9	**82.4**	14.7
10. Speak with a fast rate	5.9	**61.8**	32.4	11.8	**85.3** ^a^	2.9	2.9	**82.4**	14.7
11. Converse in an environment with air pollution	0.0	**91.2**	8.8	2.9	**97.1** ^a^	0.0	2.9	**94.1**	2.9
12. Throat clearing	61.8	**20.6**	17.6	41.2	**55.9** ^a^	2.9	38.2	**47.1**	14.7
13. Imitate car engine sounds, monster sounds	2.9	**52.9**	44.1	26.5	**50.0**	23.5	23.5	**70.6** ^b^	5.9
14. Scream	0.0	**100.0**	0.0	14.7	**76.5**	8.8	5.9	**94.1** ^b^	0.0
15. Smoke	0.0	**94.1**	5.9	0.0	**100.0** ^a^	0.0	0.0	**100.0** ^b^	0.0
16. Whispering	11.8	**38.2**	50.0	29.4	**58.8** ^a^	11.8	35.3	**55.9**	8.8
17. Breathe through the mouth	32.4	**35.3**	32.4	32.4	**61.8** ^a^	5.9	38.2	**38.2**	23.5
*Overall average (negative factors)*	*11.8*	***66.8***	*21.4*	*16.6*	***76.7***	*6.7*	*15.8*	***74.9***	*9.4*

*Note*: All items were rearranged and divided into positive and negative factors accordingly.

^a^Score is either the same or higher than the pre‐training score.

^b^Score is either the same or higher than the post‐training score.

**TABLE 5:** Distribution of responses (in percentage) for satisfaction survey taken after the training (*N* = 35).

Questionnaire item	Strongly agree	Agree	Neutral	Disagree	Strongly disagree
1. The program helps me understand skills of voice care.	**65.7**	28.6	2.9	0.0	2.9
2. The program makes me more cheerful.	45.7	45.7	8.6	0.0	0.0
3. I feel that I am healthy now.	25.7	**54.3**	20.0	0.0	0.0
4. I enjoy the activities of the program.	**60.0**	40.0	0.0	0.0	0.0
5. I can generalise the learnt knowledge into daily life.	**51.4**	45.7	2.9	0.0	0.0
6. The program widens my horizon.	**68.7**	28.6	2.9	0.0	0.0
7. Overall, I like the program.	**60.0**	40.0	0.0	0.0	0.0

	Yes	No			
8. I would like the centre to organise the program again next year.	**97.1**	2.9			

*Note*: Percentage ≥ 50% are highlighted in bold typeface.

In order to evaluate the effectiveness of the program on individual participants, a more detailed evaluation of test performance of each participant at the three different time points was done. Immediately after training, 21 out of 34 participants (61.76%) showed an increase in their post‐training scores, 5 participants (14.70%) maintained the same voice care knowledge scores as their pre‐training scores, and 8 participants (23.53%) demonstrated a decrease in their post‐training scores. When comparing the participants’ individual performance immediately after training to their performance 1 year after the training, 13 out of 34 participants (38.24%) showed an increase in their voice care knowledge scores, 9 participants (26.47%) remained unchanged, and 12 participants (35.29%) reported a decrease in their voice care knowledge scores. Among participants who reported an increase in their voice care knowledge scores immediately after training, 10 out of 21 participants (47.62%) either exhibited an additional increase in their scores or remained unchanged 1 year after the training.

In terms of degree of satisfaction towards the program, on average, the vast majority of participants (*N* = 33, 94.3%) expressed high levels of satisfaction for each evaluative item on a 5‐point scale, as indicated by their ratings of ‘strongly agree’ and ‘agree’. Additionally, 34 out of 35 participants (97.1%) expressed desire for the program to be organised again next year.

### 3.2 Qualitative Results

In total, 10 out of 34 participants (2 males, 8 females) with mean age of 66.5 years (SD = 4.88; range = 55 to 71 years) participated in the focus group interview. A total of 181 participants' statements were collected, which consisted of participants’ own words as they responded to the interview questions. The length of the statements varied from short phrases to a single paragraph. The statements were coded into 47 codes. They were later categorised into eight sub‐themes that comprised three main themes. The three themes were (1) implementation of voice care knowledge, (2) learning facilitators of the program, and (3) learning barriers and suggestions. Table 6 describes qualitative content analysis with illustrative quotes.

**TABLE 6:** Description of qualitative content analysis with examples of meaning units, condensed meaning units, sub‐themes and themes.

Meaning unit	Codes from condensed meaning units	Sub‐theme	Theme
‘I have learnt to avoid eating too many spicy and fried foods.’	Avoid eating spicy and fried food	Daily vocal hygiene practice	Implementation of voice care knowledge
‘After attending the class, I realised it was important to protect my throat. Whenever I want to cough forcefully, I will try to control myself (not to cough forcefully).’	Avoid forceful coughing		
‘(I) more or less learn something…about how to enhance my voice quality. Avoid excessive talking (because) it will harm the vocal function.’	Avoid talking without breaks		
‘I drink water as I know it is beneficial to my body. Now I have learnt that it is also beneficial to my throat. So I drink more water regularly.’	Drink more water		
‘After joining the program, I learned that ageing deteriorated my vocal cords resulting in hoarseness. It is unavoidable. But I know I shouldn't…when my voice sounds hoarse, I used to talk aloud so that others could hear me clearly. The louder I speak, the hoarser my voice would be. Now I know that I shouldn't speak too loudly as it will strain my vocal cords.’	Reduce voice volume		
‘I have learnt that ageing caused vocal folds stiffness or laryngeal deterioration, resulting in a hoarse and low‐pitched voice. I experience these vocal changes.’	Enhancement in voice care knowledge		
‘Sometimes I will educate my friends when we are chatting. I tell them to drink warm water and avoid throat clearing as it will hurt our vocal folds, reducing the voice quality.’	Spread of knowledge		
‘I only know that diaphragmatic breathing brings medical benefits and relaxes people. I have never known that diaphragmatic breathing could bring benefits to the laryngeal muscles and vocal folds to slow down its deterioration rate. Now I have learnt (its benefits) and I do it more frequently.’	Diaphragmatic breathing	Vocal facilitative techniques and exercises	
‘Sometimes I practice the phonation (exercise) when I wake up. Not always but just occasionally.’	Phonation exercise		
‘She (the speaker) used images when she was introducing (the laryngeal mechanism). For example, when we speak loudly or swallow, something covers (the airway) as barriers to protect our voices. In other words, it's harder to let things get into the airway. We referred to the image and absorbed what she was teaching all the time.’	Use of images	Learning on laryngeal mechanism	Learning facilitators of the program
‘There were some models shown to us in class so we know how they (vocal folds) open and close, how to protect them and thus we absorbed more.’	Use of tools		
‘I think short videos are easier to remember. Because they…we could read text which we tended to forget quickly. But the videos provided visual reminders and explanations. When combined with the textual information, the videos enhanced our memory retention.’	Use of videos		
‘And then he took us to the University of Hong Kong to try. Each person in our group was standing there to experience the vibration. Every person's vibration frequency was different. Some vibrations were large but some weren't. Your classmates explained to us why it was like that. Then each of us was lying on the bed to try. We didn't know what to expect and we thought that the vibrations would be exactly the same for everyone but they weren't. It's like when you attend a class to practice /a/ (to feel the vibration) for so many times. Finally, through first‐hand experience, we realized how it feels.”	Laboratory visits		
‘Because you asked us to do in‐class practice, like how to do it and then experience the differences in phonation. I didn't think there were significant differences. But it had significant impacts on vocal muscles. This is something I didn't know before.’	In‐class practice	Learning on vocal hygiene concepts	
‘The university instruments have shown us how intense and violet one's frequencies could be. By observing these instruments and vibrators, I have gained knowledge and learned to avoid speaking too loudly.’	Laboratory visits		
‘It wasn't difficult to apply it because we already knew how to do it. We were taught during class. We tried and practiced them, so there weren't any problems.’	In‐class practice	Application of vocal hygiene facilitative techniques	Learning facilitators of the program (cont’)
‘Your classmates taught us how to do, how to phonate and how to breathe during class. Those were very helpful. Sometimes, the visuals might not be clear but receiving direct on‐site instructions helped us to absorb the knowledge better. We have learned how to apply the diaphragmatic breathing and control our voice. We practiced the technique and received feedback from them to know whether we have done it correctly or not.’	Student assistance		
‘They taught us to do some relaxation exercise to relax. They showed us some videos as visual aids.’	Use of videos		
‘Sometimes, I have trouble recalling the knowledge. Whenever I want to apply them, I wonder if I have applied them correctly. I sometimes forget things.’	Memory problems	Individual difficulties	Barriers and suggestions
‘I seldom read the notes proactively. (I will) Read once a month or only when I am very free. We are not young anymore.’	Lack of motivation		
‘Talking about throat clearing, I have problems with my respiratory tract these days so I unintentionally clear my throat. Even though I have learnt not to do so, it is physically impossible and uncontrollable (to avoid throat clearing)’.	Difficulty in breaking bad habit		
‘It wasn't difficult at all. The professor and your classmates were very dedicated in teaching us so there weren't any major difficulties. It's just that when we age, we absorb and digest information slowly. While others could understand the concept after one explanation, we might need it to be explained twice to fully acquire it.’	Comprehension problems		
‘After a period of time, professionals could come and explain the key points to us again. We need not go through everything all over again unless there are new updates in voice care knowledge for us to learn’.	Need for follow‐up revision sessions	Suggestions	Barriers and suggestions (cont’)
‘(I want) Practice on our own after watching the demonstration. The more in‐class practice we have, the more we can improve.’	Need for more in‐class practice		
‘Besides everyday life tips, are there any other methods to improve our vocal quality? For example, if we have issues with our vocal folds, what should we do to address them? Also, some professionals like teachers have to speak constantly, leading to vocal polyps and vocal nodules. If one needs to undergo surgery, how can they protect their voices afterwards? Learning more about these is helpful in our daily lives. Furthermore, while we have a basic understanding of vocal production, are there any deeper insights that enable us to utilize our voice more efficiently and achieve better vocal control?’	Need for in‐depth content		
‘We had a lot of doubts. It was a rare opportunity to have the professor and your classmates there. Even it might seem like a silly question to them, it could be a significant concern for us. It will be great to receive your answers to address our concerns. I think more questions‐and‐answers sessions could be added for us to raise more questions.’	Need for more questions‐and‐answers sessions		

#### 3.2.1 Core Theme 1: Implementation of Voice Care Knowledge

##### 3.2.1.1 Vocal Hygiene Practice

Most participants reported increased awareness of the positive outcomes associated with vocal hygiene concepts. Some participants reported that the understanding of laryngeal mechanism and the concept of presbyphonia motivated them to incorporate vocal hygiene practice in their daily lives. All participants reported implementation of vocal hygiene practice proactively in a variety of activities. In everyday diet, one participant mentioned avoiding spicy and fried food, while four participants mentioned drinking more water frequently to stay hydrated. Three participants acknowledged the negative impacts brought by forceful coughing to strain the vocal folds and thus reported avoidance of forceful coughing. In conversation, seven participants reporting reduction in voice volume, with two mentioning avoiding talking without breaks to protect the voice. The implementation of voice volume reduction was extended to other contexts, such as dictation for grandchildren and playing mahjong with friends, as reported by two participants, respectively.

The positive outcomes associated with vocal hygiene had motivated several participants to perform vocal hygiene practice and spread their knowledge and experience with others. Half of the participants, who reported experiencing fewer instances of vocal fatigue and discomfort, demonstrated the initiation to educate significant others to drink more water, avoid throat‐clearing and reduce voice volume in conversation and at work. It was evident that most participants conveyed a positive attitude in implementing vocal hygiene practices widely in their daily lives.

##### 3.2.1.2 Vocal Facilitative Techniques and Exercises

More than half of the participants hold an open‐minded attitude towards practising the techniques in a range of daily activities. To exemplify, four participants reported enhanced awareness of positive outcomes associated with diaphragmatic breathing to provide additional vocal support for efficient vocal production. They reported frequent incorporation of diaphragmatic breathing in conversation and singing. Two of them generalised the use of diaphragmatic breathing across a range of contexts, such as yoga and meditation, to calm oneself down. There were a few reports of intermittent implementation of the vocal facilitative techniques. One participant demonstrated interest in using diaphragmatic breathing in singing and hiking, as well as mentioning it with friends in conversation. Two participants showed interest in doing the phonation exercises occasionally in the morning.

#### 3.2.2 Core Theme 2: Learning Facilitators of the Program

Half of the participants provided positive feedback on the content of the courses in the program, stating that it was ‘easy’, ‘detailed’ and ‘fundamental’. Three participants expressed satisfaction with the clarity of speaker's explanation throughout the program. Regarding learning on laryngeal mechanism, more than half of the participants expressed satisfaction with the effective use of visual aids, such as images, tools and videos to illustrate the laryngeal physiology clearly. Specifically, one participant reported that the laboratory visit enriched her understanding of vocal fold vibration by allowing her to observe and interact with different assessment tools. Regarding the learning of vocal hygiene concepts, one participant appreciated in‐class practice for allowing her to compare her voice condition before and after implementing various vocal hygiene practices. Another participant appreciated the opportunity to observe voice analysis procedures in the laboratory visit to deepen his understanding of the role of vocal hygiene practice in preserving vocal health. Lastly, regarding the use of vocal facilitative technique, one participant appreciated the additional hands‐on assistance by student helpers to help her familiarise herself with the technique step by step. Two participants highlighted that clear demonstration by the speaker, with the aid of videos as well as in‐class practice, helped them acquire the techniques.

#### 3.2.3 Core Theme 3: Learning Barriers and Suggestions

There were several reports of individual difficulties in learning and application of voice care knowledge. Half of the participants struggled with memory retention. They reported memory problems, leading to difficulty with retaining the voice care knowledge and recalling the vocal facilitative techniques a few days after the program. The uncertainty about executing the technique correctly hindered them from applying it frequently in daily life. Moreover, two participants reported lack of motivation to revise the knowledge and practice the techniques regularly, in which one participant commented, ‘I seldom read the notes proactively. (I will) Read once a month or only when I am very free. We are not young anymore.’ Lastly, there was single report of having difficulty in breaking the bad habit of excessive throat clearing. One participant commented that it was ‘physically impossible and uncontrollable (to avoid throat clearing)’. Another participant reported difficulty in digesting all the information, despite expressing that the material was appropriate considering their age group.

Several participants gave valid suggestions to address their memory problems and lack of motivation. First, four participants requested more in‐class practice and additional home practice for skills consolidation. For instance, one participant suggested incorporating simple and short home practice into their daily routine so as to ‘build up a linkage’ between the class and home environments, leading to greater generalisation. The addition of extra follow‐up sessions was suggested by four participants to reinforce their learning, address their questions and review the most up‐to‐date techniques. Specifically, two participants emphasised that the use of a worksheet highlighting the key points allowed them to recap the knowledge quickly. Lastly, in response to participants’ desire for more individualised support, one participant requested more question‐and‐answer sessions to address her individual concerns on vocal health while another requested for inclusion of more in‐depth content such as treatment methods for different vocal pathologies in the program.

## 4 Discussion

The purpose of the study was to evaluate the immediate effects and 1‐year maintenance of a voice education program on enhancing older adults’ voice care knowledge. The results are consistent with the hypothesis that the program could effectively enhance older adults’ voice care knowledge. Significant improvements in participants’ voice care knowledge immediately after training were observed. Improvement of some items was maintained 1 year after the training. Most participants identified various facilitators in the program that contributed to their learning experience on several topics. These facilitators included effective use of educational materials, detailed explanation, clear demonstration, in‐class practice and opportunities for laboratory visits. The enhancement in voice care knowledge had brought a positive impact on their attitudes in implementing the voice care practice in their daily lives. All participants reported integration of vocal hygiene practice, while some mentioned application of vocal facilitative techniques in their daily lives. However, the improvements in identifying a number of positive and negative factors were not maintained over a year. Some participants identified memory problems and a lack of motivation to practice and revise as the significant barriers hindering their learning process.

Surprisingly, the results revealed that participants were not well‐equipped with voice care knowledge, particularly regarding negative factors that harm the voice. Less than half of the participants could correctly identify the negative factors, such as throat clearing (20.6% accuracy), whispering (38.2% accuracy) and breathing through the mouth (35.3% accuracy) before training. A positive factor of ‘avoid chatting in noisy environment’ was correctly identified by only 38.2% of participants before training. The maintenance effect was not noted in most of the negative factors, indicated by the regression of their accuracy score collected 1 year after training. This could be explained by participants’ difficulty in breaking the habit to adopt new vocal hygiene practice, as reported by one participant, along with the memory problems and lack of motivation to revise.

Additionally, several observations were made from the participants’ responses. The voice care knowledge questionnaire included three options for each item, namely ‘positive factor’, ‘negative factor’ and ‘not certain’. As demonstrated in Table 4, participants exhibited a decrease in uncertainty in their responses for most of the items, indicated by a notable decline in the percentage of responses for the ‘not certain’ option from pre‐training to post‐training phase. However, some participants expressed increased uncertainty toward the negative factor of ‘scream’ after training. This could possibly be due to the introduction of resonant voice and voice projection, resulting in confusion and misconception for some participants to identify ‘scream’ as a negative factor.

The qualitative analysis revealed that the gain in voice care knowledge affected older adults’ attitudes positively towards generalising the vocal hygiene practice into daily lives. Several participants made subjective statements about their improved voice‐related quality of life even 1 year after the training. These results corroborate the previous literature to support the efficacy of vocal education and vocal function exercises on enhancing the voice‐related quality of life in older adults with presbyphonia (Berg et al. 2008).

Given the high prevalence rate of voice problems in older adults, previous research revealed that the concerns about facing social stigma, underestimating the severity of their voice problems and a lack of acceptance of voice deterioration hindered many older adults with self‐perceived voice problems from seeking professional help (Lindström et al. 2023). Due to the degenerative nature of presbyphonia, older adults are less likely to outgrow the voice problems as they age. In order to promote positive ageing, more efforts should be done to provide preventive voice education and training for older adults, especially targeting those in the young‐old age group (65–74 years) at an early stage (Wong and Ma 2021). Nevertheless, slight modifications should be made to yield greater treatment effect. More practice opportunities during and after the program are required for knowledge consolidation, as reported by several participants. Tailor‐made modifications, such as provision of more question‐and‐answer sessions, could be made according to participants’ preferences.

### 4.1 Limitations and Directions for Future Studies

Cautions should be exercised before generalising the current findings to a broader population. The present longitudinal study is limited in its research methodology. The efficacy of the voice education program was investigated mainly by questionnaires and small group interviews. A randomised controlled trial with a larger number of participants, involving a treatment group and a control group, is suggested for future studies.

Besides, recall bias could result as older adults are required to recall their memory related to voice care knowledge learned in the program over a period of time. To validate participants’ self‐reports, multiple data sources, such as participants’ acoustic and perceptual measurements, as well as caregiver reports, could be collected to reflect their voice health improvement in the future. Apart from the participants’ cognitive status, other factors that could have influenced the results, such as vocal comorbidities (presence of any reflux, neurological diseases) and level of vocal use in daily life, should be addressed in future studies.

## 5 Conclusions

In summary, this study provides preliminary results to support the use of a voice education program to enhance voice care knowledge of older adults. Given the supportive results in the present study, healthcare policy workers should explore the possibility to implement similar educational programs regularly in community centres, healthcare services or elderly care facilities, considering the possible logistical and motivational barriers for older adults.

## Ethics Statement

This study had attained ethics approval from the Human Research Ethics Committee of the University of Hong Kong (Approval number: EA210375). Informed consent was collected from all participants before they joined the program.

## Conflicts of Interest

The authors have declared that no competing interests existed at the time of publication.

## Data Availability

All data generated or analysed during this study are included in this article. Further enquiries can be directed to the corresponding author.
